# *Trans*-Ferulic Acid-4-β-Glucoside Alleviates Cold-Induced Oxidative Stress and Promotes Cold Tolerance

**DOI:** 10.3390/ijms19082321

**Published:** 2018-08-08

**Authors:** Chong Xue, Huanyu Lu, Ying Liu, Jianbin Zhang, Jiye Wang, Wenjing Luo, Wenbin Zhang, Jingyuan Chen

**Affiliations:** Department of Occupational and Environmental Health, Fourth Military Medical University, Key Laboratory of Hazard Assessment and Control in Special Operational Environment, Ministry of Education, Xi’an 710032, China; xuechong456@fmmu.edu.cn (C.X.); luhuanyu66@fmmu.edu.cn (H.L.); liuying2015@fmmu.edu.cn (Y.L.); zjbin777@fmmu.edu.cn (J.Z.); wangjiye@fmmu.edu.cn (J.W.); luowenj@fmmu.edu.cn (W.L.)

**Keywords:** ferulic acid, cold acclimatization, brown adipose tissue, oxidative stress

## Abstract

*Trans*-ferulic acid-4-β-glucoside (C_16_H_20_O_9_, TFA-4β-G) is a monomer extracted from the Chinese medicine called radix aconiti carmichaeli (Fuzi). To date, research on this substance is lacking. Here, we found that *trans*-ferulic acid-4-β-glucoside effectively promoted cold acclimatization in mice via increased heat production and alleviation of oxidative stress in a cold environment. Thus, our work indicates that ferulic acid-4-β-glucoside is a potential therapeutic candidate for prevention and treatment of cold stress injury.

## 1. Introduction

When exposed to a cold environment, the body activates metabolic organs through a series of different metabolic pathways to increase heat production and reduce heat loss, thus maintaining a relatively constant temperature and protecting the cells from cold stress injury. Generally, short-term cold exposure firstly induces skeletal muscle tremors to increase energy expenditure. By shivering, heat production can increase by five times compared with the baseline level [1,2]. Besides shivering thermogenesis, non-shivering thermogenesis (NST) is essential for increasing energy expenditure, especially during long-term cold exposure. Non-shivering thermogenesis causes higher energy expenditure mediated by burning brown adipose tissue and increasing mitochondrial uncoupled protein 1 (UCP1), gluconeogenesis, glycogen consumption in liver, and so on [3]. Furthermore, white adipose tissue stores triglycerides in the form of large fat droplets to increase energy storage. Conversely, with a multi-lacunar vesicle structure, brown adipose tissue has many more mitochondria and highly expresses UCP1 protein, thus uncoupling the oxidative phosphorylation pathway to interrupt ATP synthesis and releasing a large amount of energy to produce heat [4,5]. Moreover, liver—one of the most important organs in the human body—is largely involved in the metabolic process. Hepatic glucose production accounts for 90% of the endogenous glucose production [6], which is critically important to maintain the balance of glucose metabolism. Gluconeogenesis is a process that contributes to maintaining blood glucose homeostasis: the liver transforms glycerol, glycogenic amino acid, and other non-glucose substances into glucose, and this process is closely related to liver lipolysis [7].

Prolonged or intermittent repeated exposure to low temperatures enhances the body’s ability to acclimate to cold, meaning that the proportion of shivering thermogenesis decreases, while the proportion of non-shivering thermogenesis increases [8]. In 1961, Davis found the phenomenon in the human body: Prolonged regular cold exposure raised the level of non-shivering thermogenesis and resulted in cold adaptation [9]. One of the characteristics of cold acclimation is the increase of heat production induced by cold, usually accompanied with higher skin temperature but with relatively normal rectal temperature [10].

Oxidative stress is a common phenomenon that occurs in organisms faced with certain external stimuli. Once oxidation and antioxidation are out of the homeostatic state, reactive oxygen species (ROS) are overproduced. Excessive ROS—i.e., beyond the cell’s antioxidation capacity—causes oxidative stress in cells [11], and even causes other metabolic-related diseases [12]. Short-term exposure to cold can induce oxidative stress in warm-blooded mammals [13,14]. Lomakina found that the level of MDA (malondialdehyde) in the brain increased after exposure to cold for 24 h in rats [15]. In addition, Kaushik and Kaur found that rats’ brain, heart, liver, kidney, and small intestine emerged from oxidative stress after exposure to 7–8 °C with their levels of MDA increased significantly [16]. Other studies found that exposure to cold for 2 days could significantly raise HPS (hydroperoxides) levels in rats’ liver, skeletal muscle, and myocardium [17]. All these results indicate that cold exposure causes oxidative stress in many organs, further revealing that oxidative stress is widespread after cold stimulation.

The Chinese medicine radix aconiti carmichaeli (Fuzi) promotes sweating and defends the body from cold. However, the underlying mechanism remains unclear. To determine the key substance involved, we conducted purification experiments and obtained *trans*-ferulic acid-4-β-glucoside (C_16_H_20_O_9_), which has been relatively little studied. Our research found that *trans*-ferulic acid-4-β-glucoside promotes energy consumption and improves cold tolerance under low-temperature (4 °C) conditions. Furthermore, this substance may mainly be involved in liver and adipose tissue metabolism. *Trans*-ferulic acid-4-β-glucoside can significantly prevent cold-induced oxidative stress in liver and upregulate the protein level of UCP1 in brown fat tissue.

## 2. Results

### 2.1. Trans-Ferulic Acid-4-β-Glucoside Improves Cold Tolerance in Mice under Cold (4 °C) Condition

**Figure 1:**
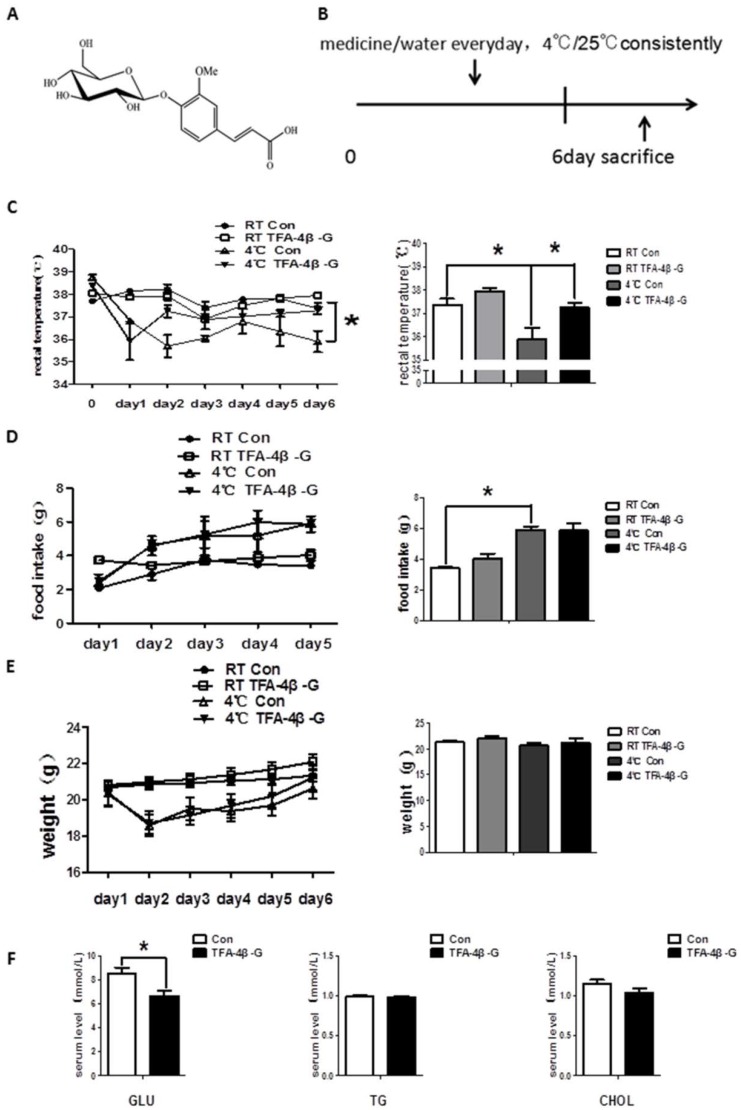
*Trans*-ferulic acid-4-β-glucoside treatment results in sustained, relatively high rectal temperature under cold conditions. (**A**) Molecular formula of TFA-4β-G; (**B**) establishment of the animal model; (**C**) change in rectal temperature among four groups (*n* = 6); (**D**) change in food intake; (**E**) change in body weight; (**F**) glucose, triglyceride, and cholesterol in serum. * *p* < 0.05 as indicated.

We established an animal model (Figure 1A,B) and found that the rectal temperature of mice exposed to cold decreased and was maintained at a relatively low level with great fluctuation. Mice treated with *trans*-ferulic acid-4-β-glucoside had a greatly increased rectal temperature, which was maintained at a relatively high level in a cold environment (Figure 1C, *p* < 0.05). Long-term cold exposure dramatically increased food intake in untreated mice (Figure 1D, *p* < 0.05). However, there was no difference in food intake between mice treated with *trans*-ferulic acid-4-β-glucoside and the control group (Figure 1D,E, *p* > 0.05). The body weight and blood biochemical parameters did not significantly alter, indicating that *trans*-ferulic acid-4-β-glucoside had no toxicity toward the liver and kidneys (Figure 1F and Figure S1F, *p* > 0.05). Intermittent-cold-exposed mice with this treatment showed the same trend (Figure S1A–D).

### 2.2. Trans-Ferulic Acid-4-β-Glucoside Alleviates Cold-Induced Oxidative Stress in Liver

**Figure 2:**
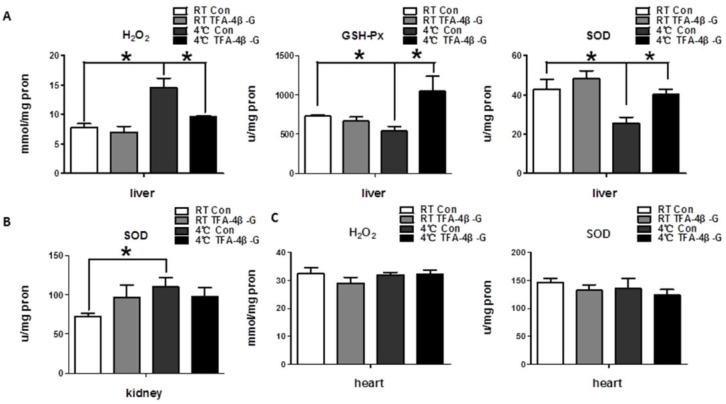
*Trans*-ferulic acid-4-β-glucoside alleviates cold-induced oxidative stress in liver. (**A**) The index related to oxidative stress in liver (*n* = 4); (**B**) the index related to oxidative stress in kidney (*n* = 4); (**C**) the index related to oxidative stress in heart (*n* = 4). * *p* < 0.05 as indicated.

The indicators of oxidative stress, ROS, include the superoxide anion (O^−^_2_), hydroxyl radical (•OH), and hydrogen peroxide (H_2_O_2_). At the same time, the organism itself can combat the occurrence of oxidative stress using an antioxidant enzyme system, including superoxide dismutase (SOD), catalase (CAT), glutathione peroxidase (GSH-Px), and so on. We detected oxidative stress injury indicators in liver, and the results revealed that long-term cold exposure increased the level of H_2_O_2_ in liver, while reducing the levels of SOD and GSH-Px. Moreover, *trans*-ferulic acid-4-β-glucoside could significantly increase the level of antioxidation, and thus alleviate oxidative stress (Figure 2A, *p* < 0.05). However, this phenomenon was not found in the heart and kidney (Figure 2B,C, *p* > 0.05).

### 2.3. Trans-Ferulic Acid-4-β-Glucoside Promotes Thermogenesis in Liver upon Cold Exposure

**Figure 3:**
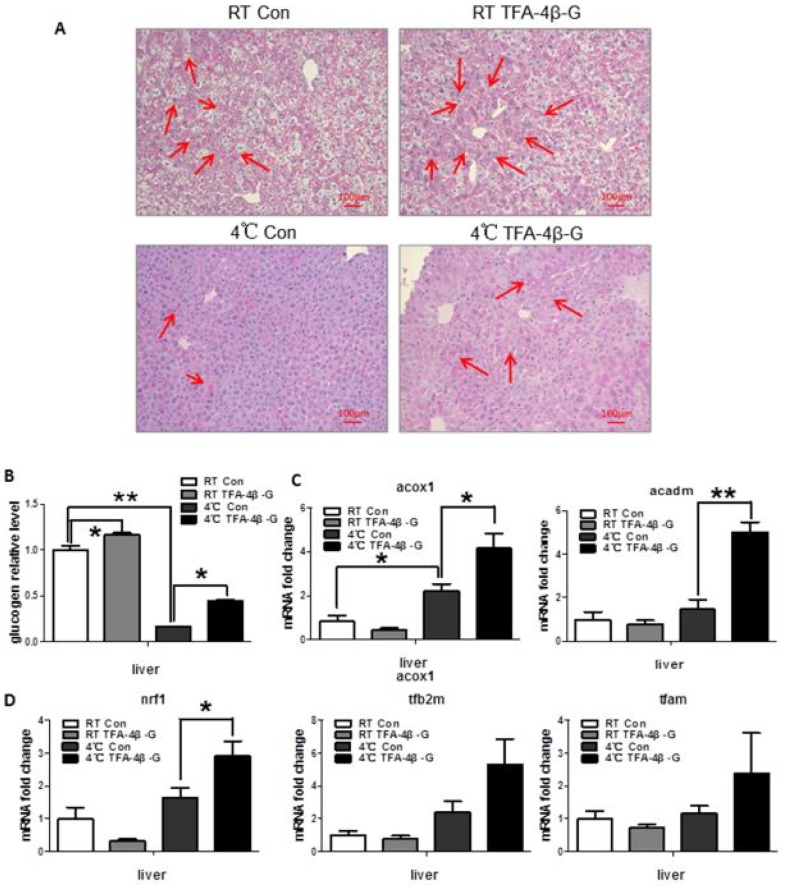
*Trans*-ferulic acid-4-β-glucoside promotes energy consumption in liver. (**A**) PAS stain in liver (red arrow indicated the glycogen); (**B**) relative glycogen expression in liver; (**C**) mRNA expression related to lipometabolism in liver (*n* = 4); (**D**) mRNA expression related to mitochondrial biogenesis in liver (*n* = 4). * *p* < 0.05, and ** *p* < 0.01 as indicated.

Hepatic glycogen content decreased under cold exposure(Figure 3B, *p* < 0.01,RT Con vs. 4°C Con), however, *trans*-ferulic acid-4-β-glucoside improved this situation (Figure 3A,B, *p* < 0.05, 4°C Con vs. 4°C TFA-4β-G). Meanwhile, the levels of genes related to mitochondrial biogenesis (the genes *nrf1*, *tfam*, and *tfb2m*) (Figure 3D, *p* < 0.05) and lipid metabolism (Figure 3C, *p* < 0.05) in the liver increased greatly after being given *trans*-ferulic acid-4-β-glucoside, according to real-time PCR results. *Trans*-ferulic acid-4-β-glucoside even increased the ATP level under cold conditions (Figure S1E, *p* < 0.05). These results suggest that *trans*-ferulic acid-4-β-glucoside increases liver heat production in mice exposed to cold (4 °C).

### 2.4. Trans-Ferulic Acid-4-β-Glucoside Activates Brown Adipose Tissue and Promotes Thermogenesis

**Figure 4:**
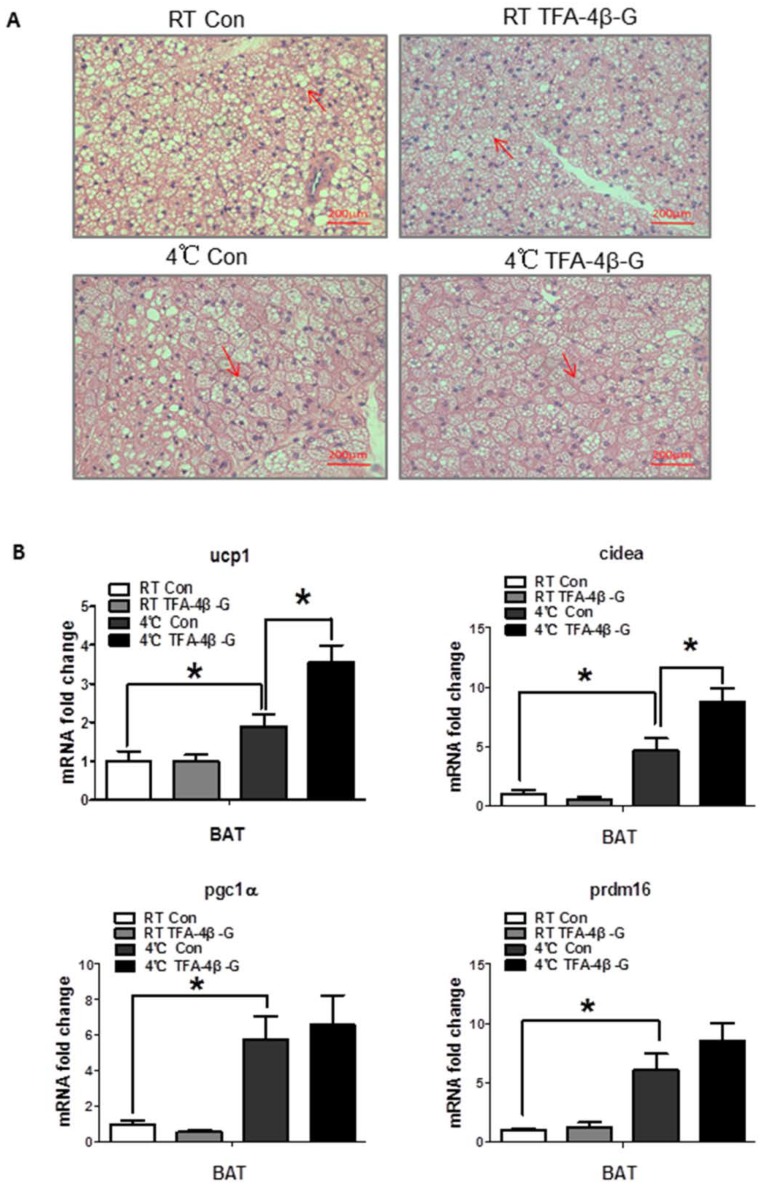

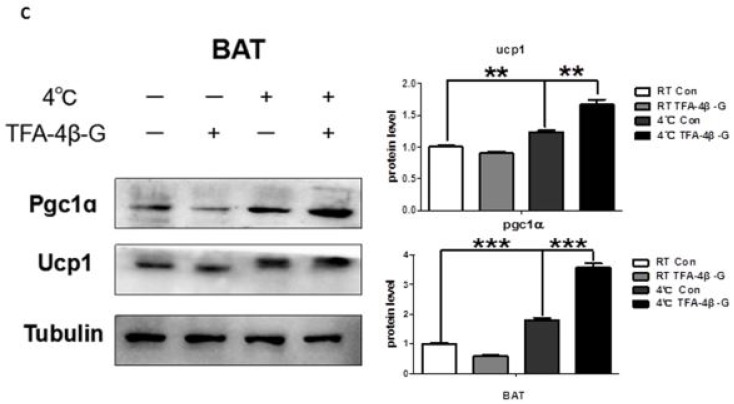
*Trans*-ferulic acid-4-β-glucoside activates brown adipose tissue. (**A**) HE stain in BAT(red arrow indicated the lipid droplets); (**B**) mRNA expression related to heat production in BAT (*n* = 4); (**C**) protein level of genes related to heat production in BAT. * *p* < 0.05, ** *p* < 0.01, and *** *p* < 0.001 as indicated.

Cold exposure increases the protein levels of UCP1 in brown adipose tissue, as well as induces thermogenesis to fight cold. The results of real-time PCR and Western blot consistently indicated that *trans*-ferulic acid-4-β-glucoside administration upregulated the expression of UCP1 in brown adipose tissue under cold conditions (Figure 4B, *p* < 0.05; Figure 4C, UCP1, *p* < 0.01; PGC1α, *p* < 0.001), which is not obvious in inguinal WAT (Figure S1I,J). At the same time, HE staining showed that the lipid droplets of the brown fat were smaller with treatment (Figure 4A). Intermittent-cold-exposed mice treated with *trans*-ferulic acid-4-β-glucoside showed the same trend (Figure S1G,H). These results suggest that *trans*-ferulic acid-4-β-glucoside promotes the activation of brown adipose tissue and increases heat production.

## 3. Discussion

As a common stimulus in the environment, cold exposure causes a series of metabolic changes in the body [18]. These changes play an important role in the formation of cold adaptation. The ability, or lack thereof, to adapt to cold determines the living conditions of mammals to a great extent, particularly in cold environments [19]. Recent studies have revealed that cold acclimation is related to the concomitant increase in brown adipose tissue (BAT) activity [8,20], as well as liver metabolism [21]. As a traditional Chinese medicine, radix aconiti carmichaeli (Fuzi) promotes heat production and protects from cold [22,23]. As the major ingredient from radix aconiti carmichaeli (Fuzi), the aconitum alkaloids mainly produce their pharmacological effects by regulating heart and blood vessel functions [24]; however, the key active ingredients from radix aconiti carmichaeli (Fuzi) which regulate BAT activity and liver metabolism in cold acclimation are still unknown. In order to elucidate the key substance that plays a role in this effect, we got a new active ingredient which is purified from Fuzi. The chemical structure is made up of ferulic acid and glucoside. Li Xia et al. found that sodium ferulate improves energy metabolism and plays a protective role after myocardial ischemia reperfusion injury, while Wu et al. suggested that ferulic acid protects the heart from lesion by inhibiting the apoptosis of mitochondria [25]. Pan Yiou et al. found that ferulic acid inhibits lipid deposition in obese mice by mediating the expression of some important lipid metabolism genes. These findings suggest that ferulic acid may participate in the process of energy metabolism and increase the body’s energy expenditure to fight obesity, however, these studies did not assess its role in liver and adipose tissue. In our study, the active ingredient *trans*-ferulic acid-4-β-glucoside promotes cold acclimation with increased fatty acid oxidation in liver and heat production in adipose tissue

By establishing and performing analysis on an animal model, we found that *trans*-ferulic acid-4-β-glucoside resulted in the maintenance of a relatively high rectal temperature level upon exposure to cold, indicating that *trans*-ferulic acid-4-β-glucoside promotes the process of cold acclimatization. Furthermore, we found that the underlying mechanism is the alleviated oxidative stress in liver and increased thermogenesis in brown adipose tissue. The genes *acox1* and *acadm* are related to fatty acid beta-oxidation and degradation of medium-chain fatty acids, respectively, and we found that *trans*-ferulic acid-4-β-glucoside increased the expression of *acox1* and *acadm* upon cold exposure, indicating the promotion of lipid oxidation. Sepa-Kishi et al. found that fatty acid oxidation was increased with cold acclimation [21], indicating that *trans*-ferulic acid-4-β-glucoside promotes cold acclimation with increased fatty acid oxidation. The genes *nrf1*, *tfam*, and *tfb2m* are related to mitochondrial biogenesis, and we found that *trans*-ferulic acid-4-β-glucoside increased the expression of these genes. Bruton et al. found stimulated mitochondrial biogenesis in muscle fibers of cold-acclimated mice [26], which also supports our results to some extent. Meanwhile, we found the expression of genes related to thermogenesis in brown adipose tissue, especially UCP1, increased with *trans*-ferulic acid-4-β-glucoside treatment, which could partially explain the improved cold tolerance in mice.

Besides, some researchers have proved that ferulic acid removes excess free radicals in the body, playing an antioxidant role. Also, sodium ferulate plays a major role in antiplatelet aggregation, neuroprotection, and so on [27]. Our study found that *trans*-ferulic acid-4-β-glucoside significantly increased antioxidant enzymes, thus reducing the oxidative stress injury in liver. For other important internal organs, such as heart and kidney, we found that the effect was not obvious. We suspect that it may be due to the following reason: As a metabolic organ, the liver may experience oxidative stress earlier than other organs, like heart or kidney. Some studies have found that acute cold exposure causes oxidative stress and organ damage in the liver of mice, but this phenomenon was not found in kidney, skeletal muscle, and brown adipose tissue [28]; which supports our conjecture.

Improved energy metabolism and alleviated oxidative stress are vital for the formation of cold acclimation [13,29]. Our results have shown that *trans*-ferulic acid-4-β-glucoside promotes cold acclimation through the above process. Some studies have focused on the relation between energy metabolism and oxidative stress and reported that oxidative stress induces autophagy in cells [30]; autophagy can reduce the damage caused by oxidative stress and thus protect cells. Meanwhile, others have reported that autophagy is closely related to energy metabolism processes, such as brown adipose tissue thermogenesis [31,32]. Therefore, whether there is a certain intrinsic link between oxidative stress and energy metabolism, and whether *trans*-ferulic acid-4-β-glucoside affects this link and thus plays a protective role, will be the next issue on molecular mechanisms to be addressed with further research.

## 4. Materials and Methods

### 4.1. Animals and Treatments

Eight-week-old C57BL/6 male mice, purchased from the experimental animal center of The Fourth Military Medical University, were kept at room temperature, with a 12 h light–dark cycle and regular chow diet. All procedures and experiments on mice were approved by Ethics Committee of Air Force Military Medical University (05 Dec 2017). We randomly divided these C57BL/6 mice into four groups to establish the animal model: one group lived at room temperature and received water, another group lived at room temperature and received *trans*-ferulic acid-4-β-glucoside, while the other two groups received water or *trans*-ferulic acid-4-β-glucoside in a cold environment (4 °C), respectively. We gave water or *trans*-ferulic acid-4-β-glucoside through intragastric administration at the fixed time 9:30 a.m. Each day, we measured the weight and food surplus before administration. After administration, we put the mice in 4 °C or room temperature environments and recorded the rectal temperature after 2 h. We repeated these operations for the next 5 consecutive days. On the 6th day, the mice were sacrificed, and the tissues were extracted and preserved at −80 °C. In addition, we established a short-time intermittent cold exposure model: We randomly divided the C57BL/6 mice into two groups, administering water or *trans*-ferulic acid-4-β-glucoside, respectively, in a 4 °C environment. The mice received cold exposure for 4 h and then were put at room temperature. The operation was repeated for 4 consecutive days. We examined the same index as mentioned above. On the 5th day, the mice were sacrificed, and the tissues were preserved.

### 4.2. Q-PCR

We used Trizol (Invitrogen, Carlsbad, CA, USA) to extract RNA from mouse liver, fat, and other tissues. With reverse transcription (TAKARA, Kusatsu, Japan), we obtained cDNA. Then, we used SYBR Green (TAKARA, Kusatsu, Japan) and the relevant primers (Sangon Biotech, Shanghai, China) to carry out real-time quantitative PCR on the FAST-7500 machine (Applied Biosystems, Foster City, CA, USA) [33]. The primer sequences are as follows: *ucp1* (sense: 5′-AGGCTTCCAGTACCATTAGGT-3′; antisense: 5′-CTGAGTGAGGCAAAGCTGATTT-3′); *pgc1α* (sense: 5′-TATGGAGTGACATAGAGTGTGCT-3′; antisense: 5′-CCACTTCAATCCACCCAGAAAG-3′); *dio2* (sense: 5′-AATTATGCCTCGGAGAAGACCG-3′; antisense: 5′-GGCAGTTGCCTAGTGAAAGGT-3′); *cidea* (sense: 5′-TGACATTCATGGGATTGCAGAC-3′; antisense: 5′-GGCCAGTTGTGATGACTAAGAC-3′); *prdm16* (sense: 5′-CCACCAGCGAGGACTTCAC-3′; antisense: 5′-GGAGGACTCTCGTAGCTCGAA-3′); *nrf1* (sense: 5′-AGCACGGAGTGACCCAAAC-3′; antisense: 5′-TGTACGTGGCTACATGGACCT-3′); *tfam* (sense: 5′-ATTCCGAAGTGTTTTTCCAGCA-3′; antisense: 5′-TCTGAAAGTTTTGCATCTGGGT-3′); *tfb2m* (sense: 5′-GGCCCATCTTGCATTCTAGGG-3′; antisense: 5′-CAGGCAACGGCTCTATATTGAAG-3′); *acox1* (sense: 5′-TAACTTCCTCACTCGAAGCCA-3′; antisense: 5′-AGTTCCATGACCCATCTCTGTC-3′); *acadm* (sense: 5′-AGGGTTTAGTTTTGAGTTGACGG-3′; antisense: 5′-CCCCGCTTTTGTCATATTCCG-3′).

### 4.3. Western Blot

The brown adipose tissue homogenate of mice was mixed with RIPA lysate (Beyotime Biotechnology, shanghai, China), protease inhibitor (Roche, Basel, Switzerland), and PMSF (Beyotime Biotechnology, shanghai, China) (100:4:1), and the mixture was placed on ice for 15 min to carry out cell lysis. Then, we centrifuged the mixture and obtained the supernatant. Next, we measured protein concentration and added 5× loading buffer (Cwbiotech, Beijing, China), boiled the sample for 7 min, and loaded the proteins for electrophoresis. After electrophoresis, we transferred the proteins to a PVDF membrane. The membrane was blocked at room temperature in 5% BSA for 2 h, and the membrane was incubated with primary antibodies (UCP1 primary antibody, rabbit 1:1000, Abcam, Cambridge, UK; PGC1α primary antibody, rabbit, 1:1000, Abcam, Cambridge, UK; ACTIN primary antibody, mouse, 1:1000, Cambridge, UK; TUBULIN primary antibody, rabbit, 1:1000, Cambridge, UK) overnight at 4 °C. The following day, the membrane was washed in TBST three times, 10 min each, and the membrane was incubated with secondary antibodies for 2 h. The protein bands were visualized by chemiluminescence (Bio-Rad, Hercules, CA, USA) [34].

### 4.4. Blood Biochemistry Analysis

Serum was obtained by centrifugation at a speed of 3000 rpm/min for 5 min. Then, blood glucose, triglycerides, and total cholesterol were measured [35], and the damage-related indexes, such as creatinine, CK (Creatine Kinase), AST (Aspartate aminotransferase), ALT (Alanine aminotransferase), and LDH (lactate dehydrogenase) were measured.

### 4.5. Liver Glycogen Staining

For conventional PAS staining (Periodic Acid-Schiff stain) [36], liver tissues fixed with 4% polyoxymethylene were paraffin-embedded, sliced, and dewaxed. Then, the slices were stained in periodate dye solution for 15 min. After washing with tap water and distilled water two times, they were stained with Schiff dye in the dark for 30 min. After washing with running water, they were stained in hematoxylin dye for 3–5 min. After differentiation, dehydration and sealing, they were observed under the microscope.

### 4.6. Brown Adipose Tissue HE Staining

Brown adipose tissue fixed by 4% polyoxymethylene was paraffin-embedded by conventional techniques [37], sliced, and dewaxed. They were stained in hematoxylin dye for 3–5 min. After washing with tap water and differentiation, they were dehydrated in 85% and 95% alcohol successively for 5 min, and stained in eosin dye for 5 min. After dehydration and sealing, they were observed under the microscope.

### 4.7. Oxidative Stress Index of Liver Tissue

T-SOD was obtained according to the T-SOD test kit protocol (Nanjing Jiancheng, Nanjing, China). Briefly, an appropriate amount of liver tissue was grinded with saline to obtain the supernatant extract. Setting up a blank tube, we mixed 0.05 mL samples of from the different groups, 1 mL reagent one and 0.1 mL each of reagent two, three, and four. Then, the mixture was placed in a water bath for 40 min at 37 °C, after which we added the chromogenic agent and measured the absorbance at a wavelength of 550 nm.

#### 4.7.1. Measurement of H_2_O_2_

H_2_O_2_ was obtained according to the H_2_O_2_ test kit protocol (Nanjing Jiancheng, Nanjing, China). Briefly, an appropriate amount of liver tissue was grinded with saline to obtain the supernatant extract. Setting up a blank tube, 0.1 mL samples or standards were mixed with 1 mL each of reagent one and reagent two, and the absorbance was measured at 405 nm wavelength.

#### 4.7.2. Measurement of GSH-px

GSH-px was obtained according to the GSH-px test kit protocol of (Nanjing Jiancheng, Nanjing, China). Briefly, an appropriate amount of liver tissue was grinded with saline to obtain the supernatant extract. A blank tube, standard tube, enzyme tube, and non-enzyme tube were set up. The reagents were mixed together according to the recommended quantities and the absorbance was measured at a wavelength of 412 nm.

### 4.8. Statistical Analyses

The data were expressed as mean ± standard error of the mean (SEM). The results were tested by one-way ANOVA with Tukey’s post hoc t tests among the groups, and the differences between the two groups were analyzed using Student’s *t*-test. *p* values <0.05 were considered statistically significant. All these analyses were performed using SPSS20.0 software.

## Supplementary Materials

The following are available online http://www.mdpi.com/1422-0067/19/8/2321/s1.

## Abbreviations

TFA-4β-G	*Trans*-ferulic acid-4-β-glucoside
NST	non-shivering thermogenesis
UCP1	uncoupled protein 1
MDA	malondialdehyde
TBARS	thiobarbituric acid-reactive substances
HPS	hydroperoxides
ROS	reactive oxygen species
