# Supplementary material for: Trans-Ferulic Acid-4-β-Glucoside Alleviates Cold-Induced Oxidative Stress and Promotes Cold Tolerance
Source: Int J Mol Sci. 2018 Aug 8;19(8):2321. doi: 10.3390/ijms19082321 (PMC6121433; doi:10.3390/ijms19082321)
Supplement: Supplementary file 1 [file ijms-19-02321-s001.docx]

**
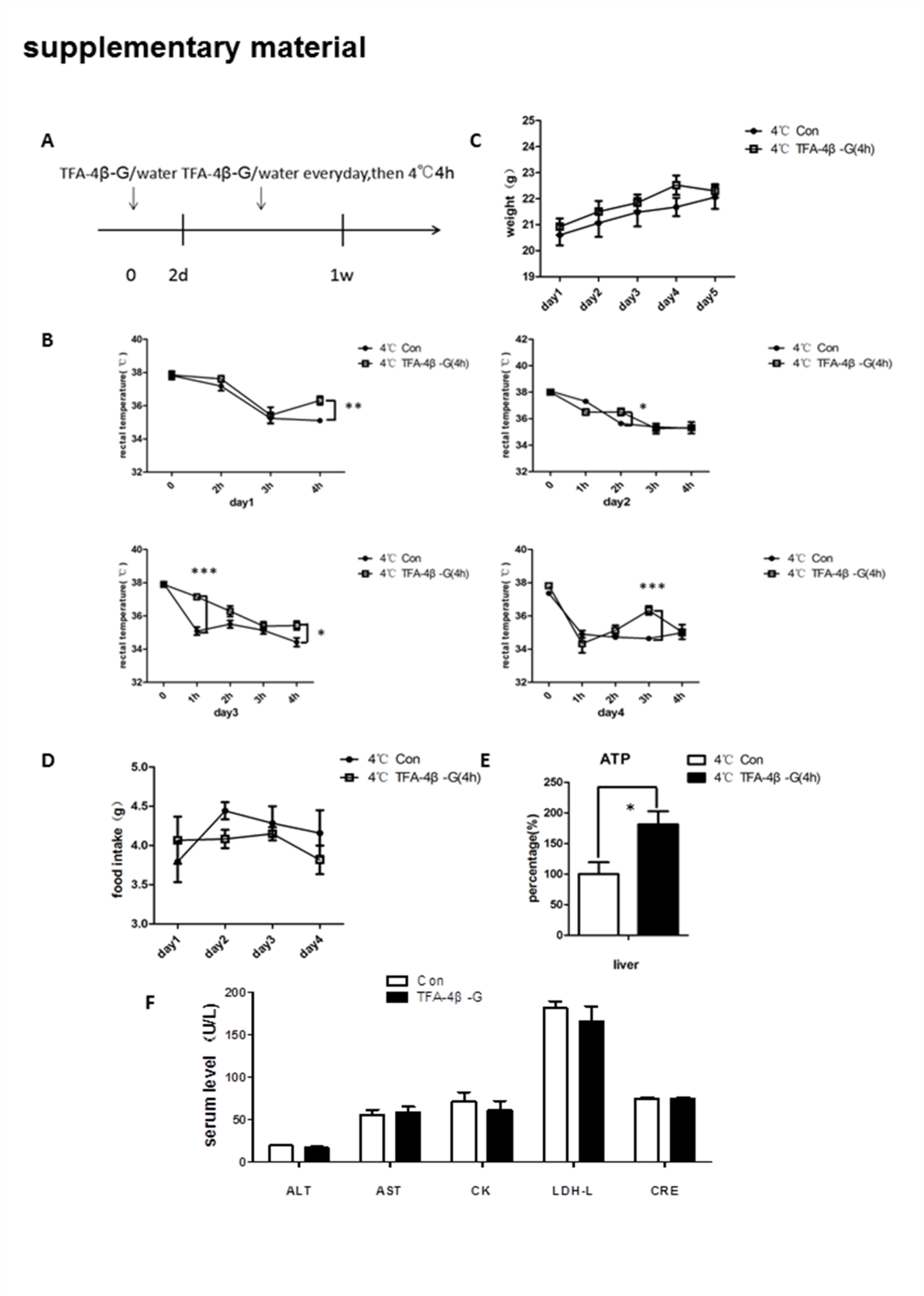
**

**
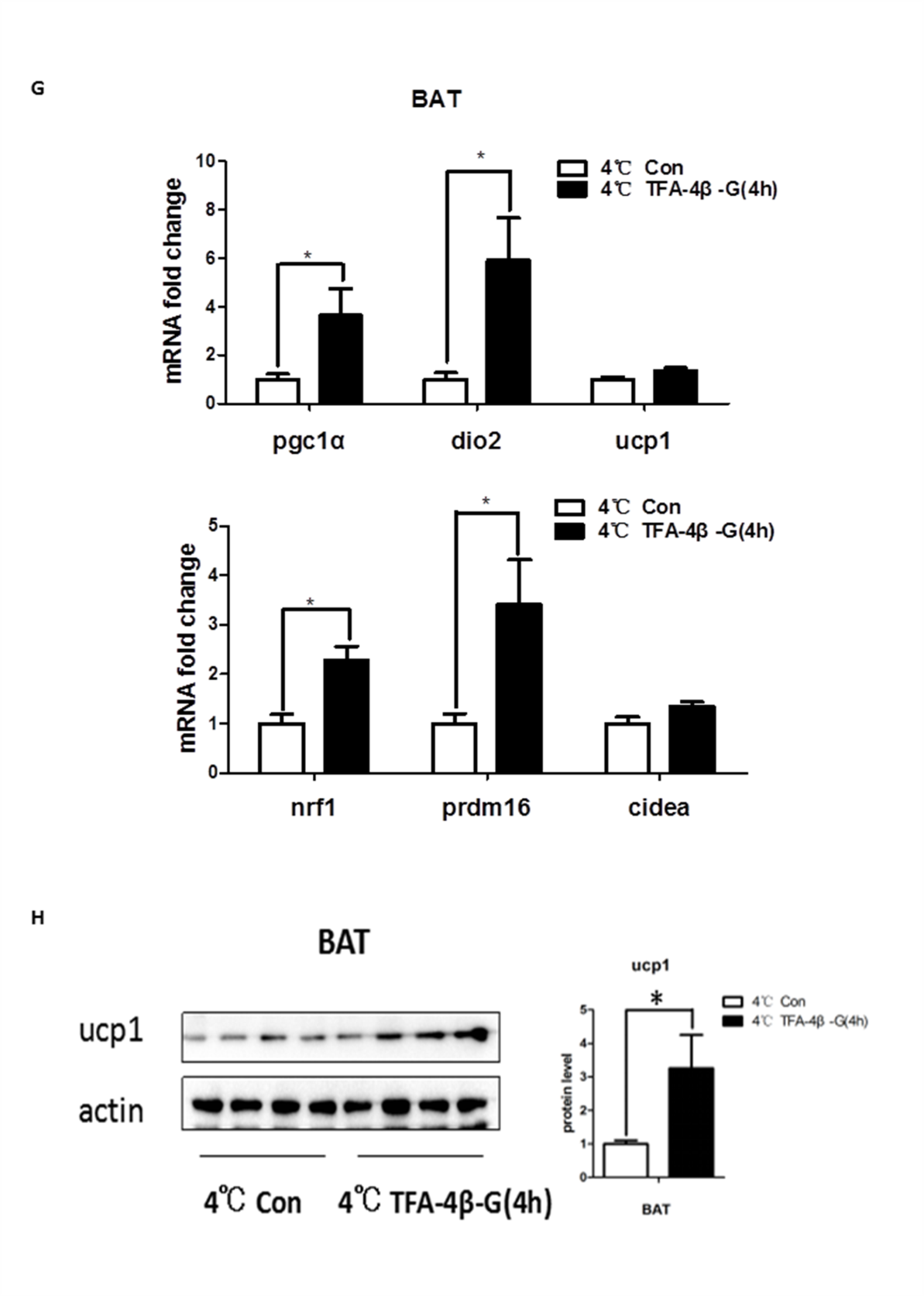
**

**
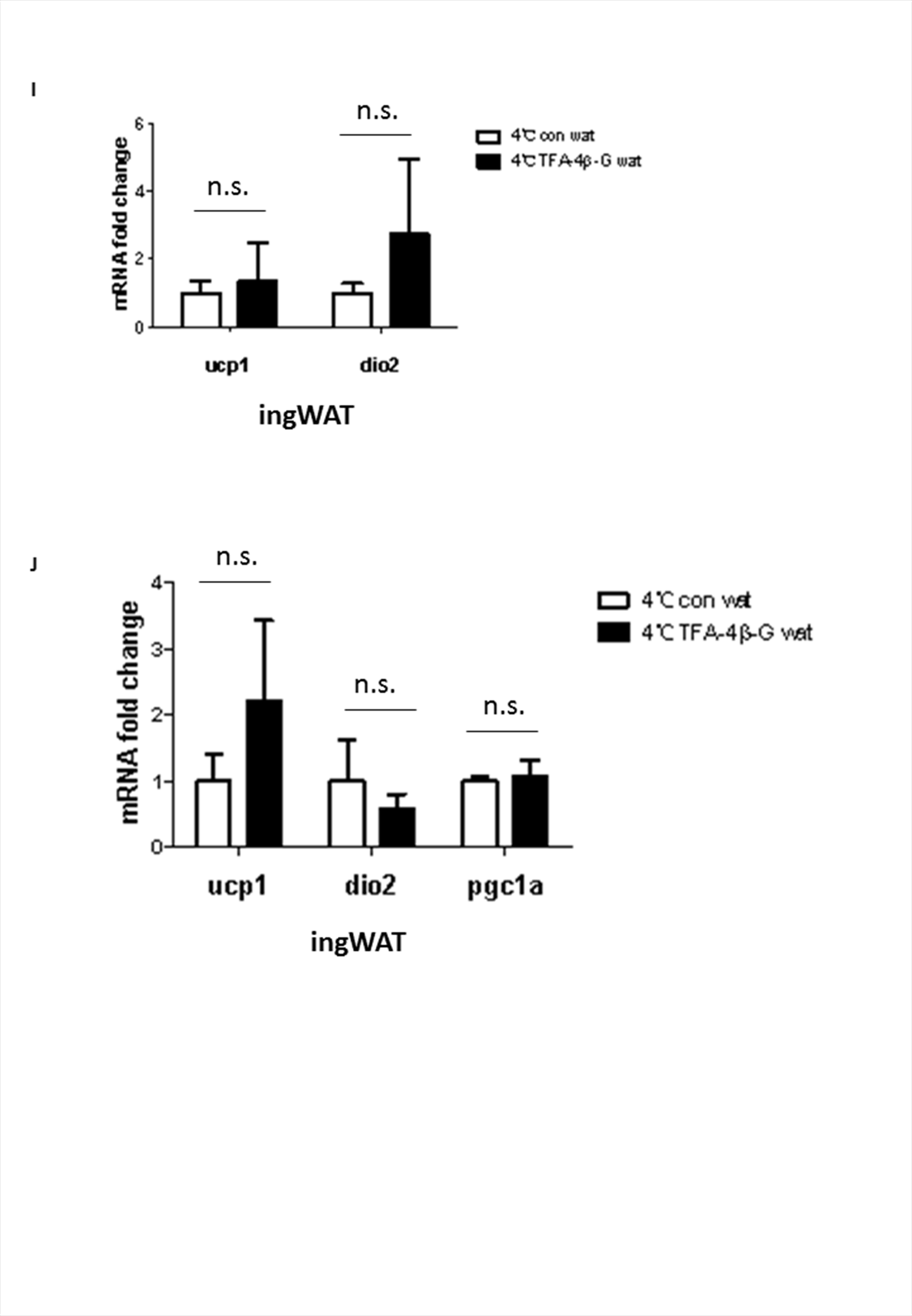
**

**Figure S1.** (**A**) The establishment of the animal model for intermittent repeated exposure to low temperature; (**B**) daily changes in rectal temperature (n = 6); (**C**) changes in body weight; (**D**) changes in food intake; (**E**) ATP level in liver; (**F**) ALT, AST, CK, LDH-L, CRE in serum; (**G**) mRNA expression related to heat production in BAT (n = 4); (**H**) protein level of UCP1 in BAT (n = 4).(I) mRNA expression related to heat production in inguinal WAT upon consistent cold exposure (n=4).(J) mRNA expression related to heat production in inguinal WAT upon intermittent cold exposure(n=4).
